# Ultrathin Fiber‐Mesh Polymer Thermistors

**DOI:** 10.1002/advs.202202312

**Published:** 2022-09-04

**Authors:** Chihiro Okutani, Tomoyuki Yokota, Takao Someya

**Affiliations:** ^1^ Department of Electrical Engineering and Information Systems The University of Tokyo 7‐3‐1 Hongo, Bunkyo‐ku Tokyo 113‐8656 Japan; ^2^ Department of Electrical and Computer Engineering Shinshu University 4‐17‐1, Wakasato Nagano City Nagano 380‐8553 Japan

**Keywords:** composite materials, fiber‐mesh structures, polymer thermistors, ultrathin electronics

## Abstract

Flexible sensors enable on‐skin and in‐body health monitoring, which require flexible thermal protection circuits to prevent overheating and operate the devices safely. Here, ultrathin fiber‐mesh polymer positive temperature coefficient (PTC) thermistors via electrospinning are developed. The fiber‐type thermistors are composed of acrylate polymer and carbon nanofibers. The fibrous composite materials are coated with a parylene to form a core–sheath structure, which improves the repeatability of temperature characteristics. Approximately 5 µm thick fiber‐type thermistors exhibit an increase in the resistance by three orders of magnitude within ≈2 °C and repeatable temperature characteristics for up to 400 cycles. The mesh structure enables the thermistor layer to be ultra‐lightweight and transparent; the mesh‐type thermistor operates with a fiber density of 16.5 µg cm^−2^, whose fiber layer has a transmittance of more than 90% in the 400–800 nm region. By fabricating the mesh thermistor on a 1.4 µm thick substrate, the thermistor operates without degradation when wrapped around a 280 µm radius needle. Furthermore, the gas‐permeable property is demonstrated by fabricating the fibrous thermistor on a mesh substrate. The proposed ultrathin mesh polymer PTC thermistors form the basis for on‐skin and implantable devices that are equipped with overheat prevention.

## Graphical abstract

An ultrathin fiber‐mesh polymer positive temperature coefficient thermistor is developed via electrospinning. The thermistor consists of ≈5 µm thick fibers and exhibits an increase in the resistance by three orders of magnitude within ≈2 °C and repeatable temperature characteristics for up to 400 cycles. The ultrathin mesh structure enables the thermistor layer to be ultra‐lightweight, transparent, flexible, and gas‐permeable.

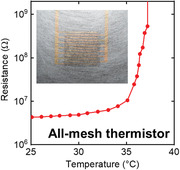

## 1 Introduction

Thin and flexible electronic devices compatible with the soft/sensitive skin of human bodies are gradually increasing. Therefore, they are now commonly utilized in healthcare^[^ ^1^, ^2^, ^3^, ^4^, ^5^, ^6^, ^7^, ^8^, ^9^, ^10^ ^]^ and medical fields.^[^ ^11^, ^12^, ^13^, ^14^ ^]^ In particular, ultrathin electronic devices can be mounted on complex shapes such as skins^[^ ^7^, ^8^, ^9^, ^10^, ^15^, ^16^, ^17^, ^18^, ^19^ ^]^ and curved surfaces.^[^ ^11^, ^18^, ^20^, ^21^ ^]^ In addition, the thinness of these devices makes them robust to mechanical strains caused by bending. Ultrathin devices with <10 µm thickness have ultraflexibility, wherein the properties do not change when crumpled or wrapped around needles or catheters‐sized several hundred micrometers.^[^ ^8^, ^21^ ^]^ Owing to such mechanical flexibility, the device remains unbroken with a wrinkled structure caused by prestretching, which induces stretchable properties.^[^ ^19^, ^22^ ^]^ Moreover, the flexibility of the device is enhanced using mesh structures that are robust to mechanical deformations.^[^ ^6^, ^7^, ^15^ ^]^ Mesh networks comprised of polymeric micro/nanofibers can be easily fabricated via electrospinning.^[^ ^23^ ^]^ The porous structure of the fiber mesh has additional benefits pertaining to inherent features, such as gas permeability and transparency.^[^ ^5^, ^6^, ^7^, ^16^, ^17^, ^24^ ^]^ Gas permeability reduces irritation and discomfort during long‐term skin attachment,^[^ ^4^, ^9^, ^15^ ^]^ and transparency eliminates the presence of the device and is important to be compatible with optical sensors. Moreover, polymer composite materials can be fabricated into fibers via electrospinning.^[^ ^24^, ^25^ ^]^

The use of on‐skin sensors for health monitoring,^[^ ^3^, ^4^, ^5^, ^6^, ^7^, ^8^, ^9^, ^10^ ^]^ inserted probes for stimulation,^[^ ^12^, ^26^ ^]^ and catheter‐mounted sensors for medical treatment^[^ ^13^, ^21^ ^]^ generates heat during operation. Therefore, an overheat protection circuit is required to prevent damage to biological tissues caused by burns from the heat generated during the operation of such devices. The development of a bendable thermal protection circuit can expand the applicability of flexible electronic devices.

The devices with a large change in resistance within a narrow temperature range serve as thermal protection circuits. One candidate is a polymer positive temperature coefficient (PTC) thermistor, which is a composite material fabricated by mixing a crystalline polymer and conductive materials.^[^ ^27^ ^]^ The thermistor has the property of increasing the resistance sharply by more than three orders of magnitude as the temperature increases.^[^ ^28^, ^29^, ^30^, ^31^, ^32^, ^33^ ^]^ The large change in resistance in a narrow temperature range has been applied in sensitive temperature sensors^[^ ^28^, ^29^, ^30^ ^]^ and self‐temperature‐controllable devices.^[^ ^31^, ^32^, ^33^ ^]^ Commercial polymer PTC thermistors are sized several hundred micrometers. Additionally, recent polymer PTC thermistors with a thin‐film structure of <20 µm thickness have been reported,^[^ ^29^, ^30^, ^31^, ^33^, ^34^ ^]^ some of which have exhibited high mechanical flexibility to operate while bent.^[^ ^29^, ^30^ ^]^ However, for thermistors to be applied for on‐skin medical sensors, they are required to be stretchable and bendable down to several hundred micrometers. Nevertheless, it is challenging to fabricate a thermistor whose temperature characteristics do not deteriorate when wrapped around a needle with a bending radius of <1 mm. In particular, ultrathin and ultraflexible thermistors fabricated with conductive materials embedded in polymer fibers have not been developed.

In this study, we developed an ultrathin mesh‐type polymer PTC thermistor using the electrospinning process. We obtained composite fibers with a thickness of ≈5 µm by dispersing carbon nanofibers in the polymer solution using the ultrasonic treatment. The fiber‐mesh thermistor exhibited an increase in resistance of three orders of magnitude in a narrow temperature range (≈2 °C) and repeatable temperature characteristics for up to 400 cycles. The mesh structure allowed the thermistor layer to be transparent (>90%) in the 400–800 nm region. Furthermore, we demonstrated the operation of the thermistor wrapped around a 280 µm needle by fabricating the composite fibers on a 1.4 µm ultrathin film. Moreover, an entirely porous PTC thermistor was developed to achieve gas permeability.

## 2 Results

**Figure 1:**
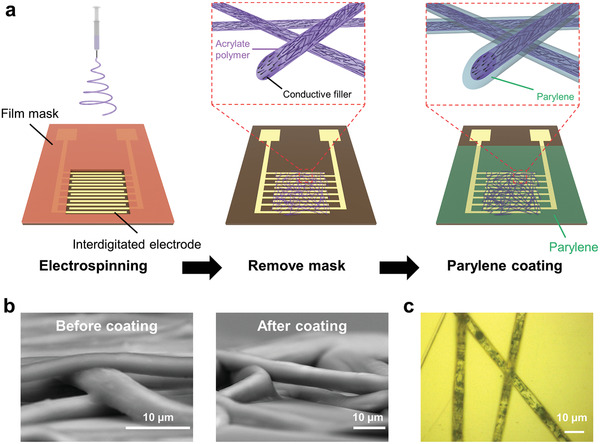
Fabrication of fiber‐mesh thermistors. a) Schematic of the fabrication process of fiber‐mesh polymer positive temperature coefficient (PTC) thermistors. b) Cross‐sectional images of composite fibers before and after parylene coating. c) Optical microscope image of composite fibers after parylene coating.

We fabricated mesh‐type polymer PTC thermistors on interdigitated electrodes via a film mask (**Figure** **1**a). The fibrous thermistors were fabricated via electrospinning using a solution of composite materials. The solution primarily comprised an acrylate polymer, which served as the polymer matrix of the fibers. The acrylate polymer was slightly modified from that used in previous studies^[^ ^29^, ^30^, ^35^ ^]^ to reduce the thickness of the fabricated fibers. In addition to octadecyl acrylate and butyl acrylate, 0.015 wt% poly(3,4‐ethylenedioxythiophene)‐tetramethacrylate (PEDOT‐TMA) was mixed for synthesis (Figure S1, Supporting Information). The addition of PEDOT‐TMA affected the electrospun fiber diameter of the pure acrylate polymer (Note S1 and Figures S2, Supporting Information). The fiber diameter of the acrylate polymer without PEDOT‐TMA was 14.6 µm, whereas that of the polymer with 0.015 wt% PEDOT‐TMA was 6.4 µm (Figures S2, Supporting Information). Unlike the differences in the fiber diameter, the addition of PEDOT‐TMA little affected the thermal property of polymers and thermal characteristics of PTC thermistors (Figures S3a,b and S4, Supporting Information). We used 6 wt% carbon nanofibers as conductive fillers, which were dispersed by sonication in a polymer solution with tetrahydrofuran (THF). Herein, the 6 wt% was determined based on the device properties of the film thermistors and the ability to fabricate fibers stably (Note S2 and Figure S5, Supporting Information). The fabricated fibrous thermistors were extremely thin with a fiber thickness of ≈4 µm according to the scanning electron microscope (SEM) images (Figure 1b and Figure S6a, Supporting Information). After fiber formation, a thin layer of parylene was coated using chemical vapor deposition. The parylene layer was deposited around the fiber, generating a core–sheath structure. This encapsulation process increased the thickness of fibers but maintained fiber‐mesh structures (Figure 1b and Figure S6b, Supporting Information). The optical microscope images indicated that the conductive fillers were dispersed in the fibers with small aggregates (Figure 1c).

**Figure 2:**
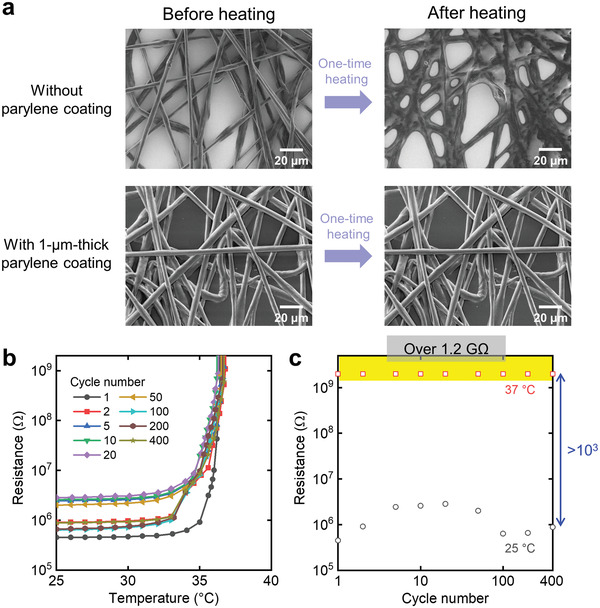
Effect of parylene coating on the thermal cyclic characteristics of the mesh thermistor. a) SEM images of composite fibers without parylene coating and with 1 µm parylene coating before and after one‐time heating in an oven at 70 °C for 5 min. b) Changes in the resistance of mesh thermistors with 1 µm parylene coating as a function of temperature. c) Reversible thermal switching behavior of a mesh thermistor with 1 µm parylene coating. Resistances at 25 and 37 °C were extracted from (b) as a function of the cycle number.

Coating the parylene layer improved the repeatability of thermal characteristics by maintaining the fiber‐mesh structure. The large change in resistance with respect to the temperature of polymer PTC thermistors can be attributed to the large change in volume caused by the melting of the polymer.^[^ ^36^ ^]^ When the fibrous thermistors were not coated with parylene, the fibers melted as the temperature increased and failed to maintain their shapes (**Figure** **2**a). However, when a 1 µm thick parylene was coated, the parylene sheath fastened the core of the composite fiber. The core–sheath structure helped maintain the shape of the fiber‐mesh thermistor even when the temperature increased beyond the melting point of the polymer matrix (Figure 2a). We investigated the thermal cyclic characteristics of the mesh thermistors with 1 µm parylene layer. The resistance of the mesh thermistors increased sharply from near 35 °C, which is consistent with the melting phenomenon of the composite material (Figure S5c, Supporting Information). The characteristics of the increase in the resistance were maintained even at the 400th temperature increase (Figure 2b). Typically, polymer PTC thermistors are expected to be applied as resettable fuses.^[^ ^31^, ^33^ ^]^ Therefore, to evaluate the thermal cyclic characteristics, we analyzed whether the mesh thermistors are capable of maintaining a high resistance value (switch‐off function) at a high temperature (37 °C) (Figure 2c). The mesh thermistors coated with 1 µm parylene maintained a resistance value of over 1.2 GΩ at 37 °C for up to 400 cycles, and the ratio of resistance change compared to that at 25 °C was nearly 10^3^. In the cycle tests, the resistance at 25 °C varied from 0.45 to 2.8 MΩ (Figure 2b,c). The variation in the initial resistance can be attributed to changes in the conductive paths by the movement of conductive fillers during temperature cycle tests. The optical microscope images indicate that the positions of the conductive fillers changed after the cyclic tests (Figure S7, Supporting Information). Unlike film‐type thermistors, mesh thermistors can possess only a limited number of conductive paths inside the fibers. This indicates that even slight movements of the conductive fillers affect the change in the conductive path significantly. Furthermore, the limited number of conductive paths affects the variation in the resistance of the thermistor. When seven mesh thermistors were fabricated simultaneously with a spinning time of 20 s, all devices operated with ideal thermistor characteristics; however, the resistances at 25 °C varied from 0.40 to 1.6 MΩ (Figure S8, Supporting Information).

**Figure 3:**
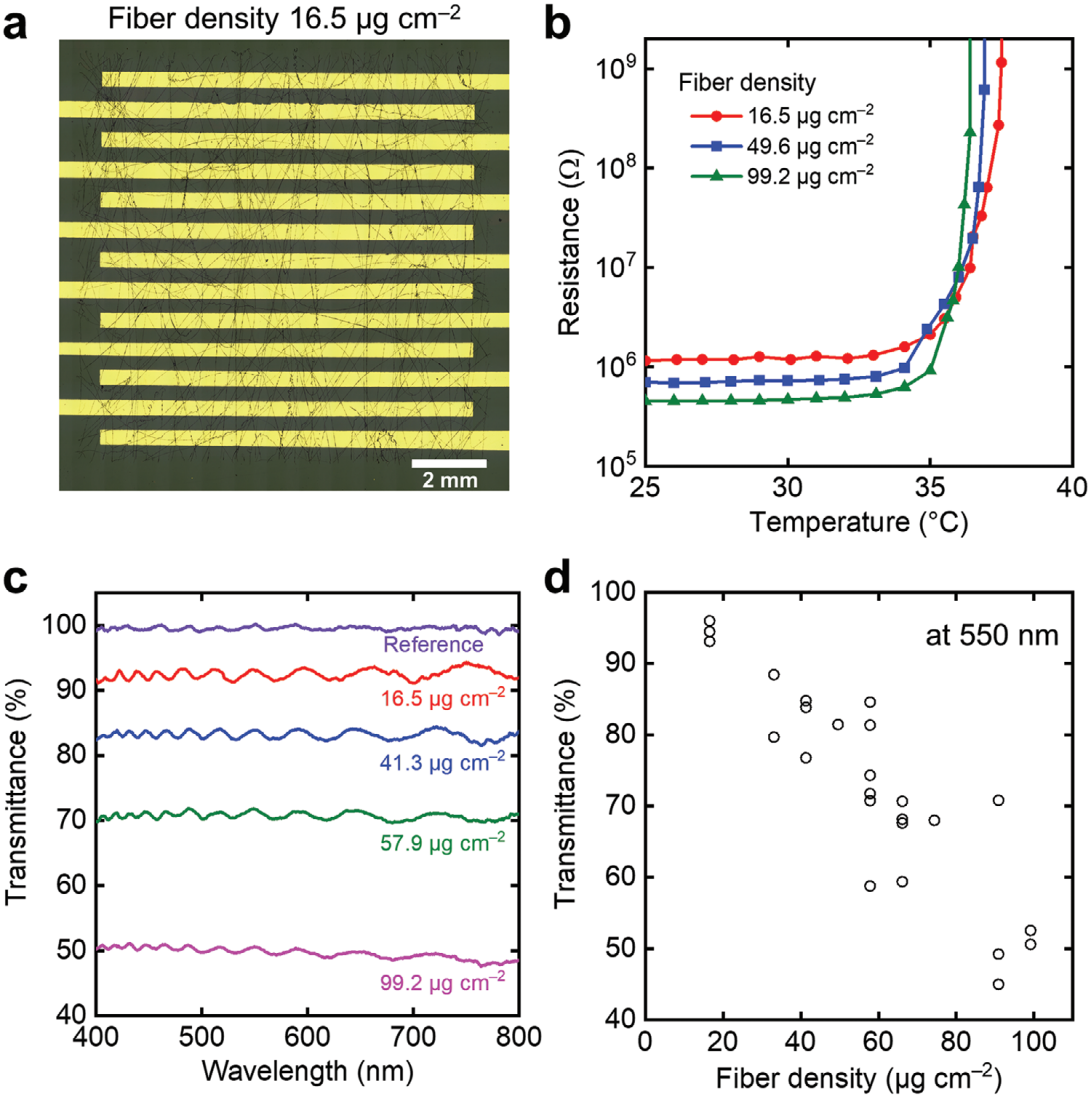
Transparency of the composite fiber layer depending on the fiber density. a) Optical image of the mesh thermistor with a density of 16.5 µg cm^−2^. b) Change in the resistance of different fiber densities as a function of temperature. c) Optical transmittance of the composite fiber layer as a function of wavelength from 400 to 800 nm for each density. d) Transmittance of the composite fiber layer at 550 nm as a function of fiber density.

The mesh structure with fibers results in porous‐derived transparency in the device.^[^ ^16^, ^24^ ^]^ Despite using black conductive fillers, interdigitated electrodes under the fiber layer were observed when the fiber layers were fabricated with a low fiber density of 16.5 µg cm^−2^ (**Figure** **3**a). Regardless of the thin fiber layer, the thermistor exhibited a change in the resistance by three orders of magnitude from ≈35 °C, which is similar to those with thick fiber layers such as 49.6 and 99.2 µg cm^−2^ (Figure 3b). Additionally, we investigated the transparency of the fiber layer with respect to its density by depositing a fiber layer on a transparent film. As the density increased, the color of the fiber layer turned black, which was derived from the color of the conductive fillers (Figure S9, Supporting Information). The transmittance decreased by nearly 10% at a density of ≈20 µg cm^−2^ (Figure 3c,d). For a fiber density of 16.5 µg cm^−2^, the fiber layer exhibited high transmittances of >90% in the 400–800 nm region, and therefore the thermistors with both the large change in resistance and transparency were realized. This value is comparable to that of a film‐type thermistor with a low loading concentration of fillers.^[^ ^30^ ^]^

**Figure 4:**
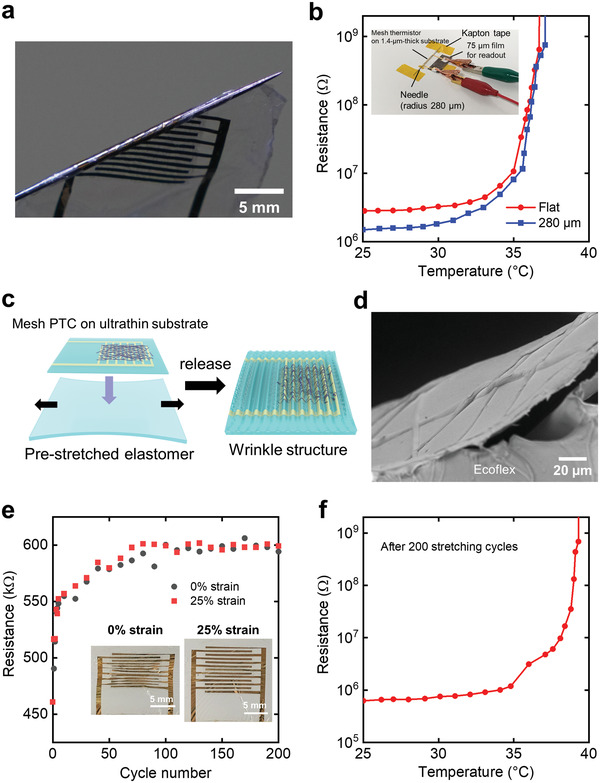
Temperature and mechanical characteristics of an ultraflexible thermistor. a) A photograph of the mesh thermistor on an ultrathin film substrate. b) Ultrathin thermistor characteristics in the flat and bent states. Inset: a photograph of the thermistor wrapped around the needle during measurement. c) Illustration of the prestretching method. d) Cross‐sectional SEM image of the wrinkled thermistor. e) Change in resistance during cyclic stretching tests. Inset: Photographs of the wrinkled thermistor at 0% and 25% strains. f) Change in the resistance of wrinkled thermistor as a function of temperature after 200 stretching tests.

Furthermore, we fabricated the composite fibers on an ultrathin substrate to obtain an ultraflexible mesh thermistor. Reducing the overall thickness of a device can significantly reduce the strain caused by bending.^[^ ^19^, ^20^, ^21^, ^37^ ^]^ The composite fibers were electrospun onto interdigitated electrodes on a 1.4 µm polyethylene terephthalate (PET) substrate that was attached to a supporting substrate. Subsequently, a 1.4 µm thick parylene layer was coated on them. An ultrathin device could be realized by peeling the device off from the supporting substrate (**Figure** **4**a). The resistance value of the thermistor changed little before and after detaching it from the supporting substrate. The mesh thermistor on the ultrathin substrate exhibited a change in resistance of less than 5% when wrapped around a needle with a bending radius of 280 µm (Figure S10a, Supporting Information). Additionally, similar to when the thermistor was placed flat, the thermistor exhibited a large change in resistance with the increase in temperature despite being wrapped around the needle (Figure 4b). Note that the difference in the resistance at 25 °C was derived from the variation, not bending. To the best of our knowledge, this mechanical durability is superior to that reported previously in the case of polymer PTC thermistors.^[^ ^29^, ^30^ ^]^ Such high durability against bending offers stretchability by forming a wrinkled structure.^[^ ^19^, ^22^ ^]^ We fabricated a wrinkle‐structured device by attaching an ultraflexible thermistor to a 25% prestretched elastomer and releasing the stretched state (Figure 4c). The fiber layer adhered to the ultrathin substrate despite being deformed into this wrinkled structure with a bending radius of several hundred micrometers (Figure 4d and Figure S10b, Supporting Information). This is due to the high flexibility of the ultrathin fibers. The mesh thermistor exhibited a large increase in resistance when the temperature increased in the compressed wrinkled state (Figure S10c, Supporting Information). Moreover, the temperature characteristics changed little when the wrinkled thermistor was stretched by 20% (Figure S10c, Supporting Information). However, the temperature at which the large resistance changes began increased by ≈2 °C. This can be attributed to the 1.2 mm thick elastomer, which complicates the warming up of the device. We also investigated the mechanical durability of wrinkled thermistors with a cyclic stretching test. Although the resistance of the thermistor increased by ≈30% after 200 cycles of 25% stretching, the thermistor characteristics of the large increase in the resistance were maintained, indicating high mechanical durability (Figure 4e,f and Figure S10d, Supporting Information).

**Figure 5:**
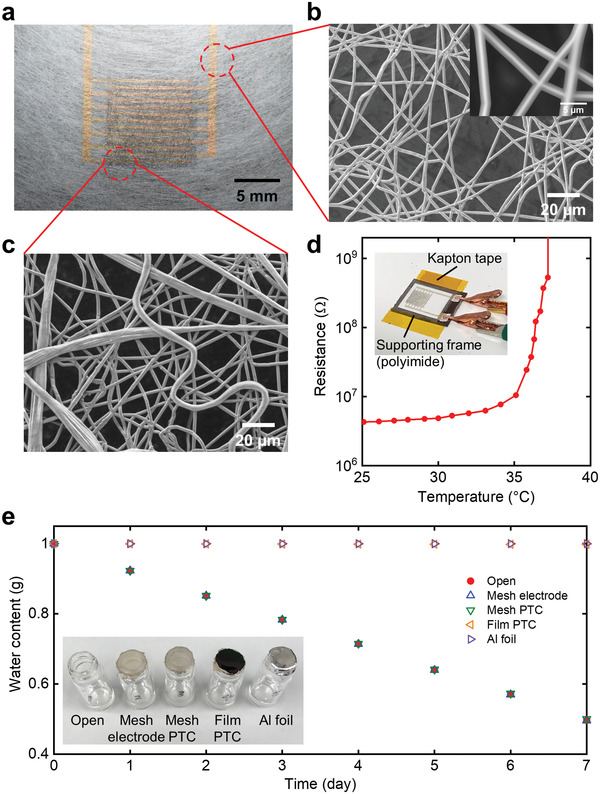
Thermal and gas‐permeable characteristics of the thermistor with all‐mesh structures. a) A photograph of the mesh thermistor on a mesh substrate. b) SEM image of the mesh substrate. Inset: a magnified image of the parylene‐coated mesh electrode at an acceleration voltage of 15 kV. c) SEM image of the parylene‐coated composite fibers on the mesh substrate. d) Change in the resistance of the thermistor with all‐mesh structures as a function of temperature. Inset: a photograph of the fiber‐type thermistor on the mesh substrate with a supporting frame during measurement. e) The subsequent water weight loss for each sample. Inset: a photograph of samples.

Finally, we obtained an all‐mesh thermistor by fabricating composite fibers on a mesh substrate (**Figure** **5**a). The mesh substrate was developed by preparing a heat‐pressed poly vinyl alcohol (PVA) fiber sheet and patterning gold electrodes on it, which was used in our previous research^[^ ^38^ ^]^ (Figure S11a, Supporting Information). However, we used a lower fiber density than that used in the previous study to retain the mesh holes after the parylene coating. Additionally, a polyimide supporting frame was used to perform the fabrication process in a freestanding state (Figure S11b, Supporting Information). The composite fibers were electrospun onto the interdigitated electrodes on the PVA mesh substrate and coated with 700 nm parylene (Figure S11c,d, Supporting Information). When the parylene‐coated mesh electrode was observed under the SEM at a high voltage of 15 kV, the parylene section turned semitransparent, which revealed the gold section (Figure 5b). This implies that the 700 nm parylene was uniformly coated around ≈700 nm wide gold electrodes. The 1 min electrospun mesh thermistor on the mesh substrate continued to be porous after the parylene coating (Figure 5c). This is because the densities of both thermistor and substrate layers are low. When fabricated on a mesh substrate, the mesh thermistors exhibited a large increase in resistance with the increase in temperature, which is similar to that observed when fabricated on a film (Figure 5d). This result validates that polymer PTC thermistors can be operated with porous structures. Furthermore, we compared the gas permeability of porous thermistors with that of film thermistors by evaluating the water vapor permeability.^[^ ^5^, ^9^, ^10^ ^]^ In the case of a film thermistor, a 25 µm thick thermistor was prepared on a mesh substrate and coated with 700 nm parylene. The film‐type thermistor exhibited 0.4% water vapor transmission in comparison with the open state, which was comparable to that of the Al foil (0%) (Figure 5e). By contrast, the all‐mesh thermistor exhibited 99.2% water vapor permeability compared to the open state. Thus, the addition of an unconventional property of gas permeability was demonstrated by fabricating the fibrous thermistor with a mesh structure rather than a film structure.

## 3 Discussions

Unlike a previous study in which electrospun fibers were coated with conductive particles and embedded in a polymer matrix,^[^ ^39^ ^]^ we generated the conductive paths inside the fibers by using sonication to disperse carbon nanofibers in a polymer solution. This process enabled the fabrication of polymer PTC thermistors with a mesh structure.

In this study, we used an acrylate polymer which allowed the thermistor to react from 35 °C (near body temperature). The reaction temperature range of thermistors can be changed either by using acrylate polymers with different melting points^[^ ^29^, ^40^ ^]^ or other crystalline polymers.^[^ ^28^, ^31^ ^]^ This can expand the range of applications such as thermal protection circuits at high temperatures.^[^ ^31^, ^32^, ^33^ ^]^

However, mesh‐type thermistors exhibit certain problems of instability in conductive path formation and a high resistance value. The instability caused the initial resistance to vary among samples and during the cyclic tests (Figure 2b,c and Figures S7 and S8, Supporting Information). The variation hinders the systematic characterization of thermistors. In this study, the characteristics of the large change in resistance in a narrow temperature range were observed, but detailed evaluations have not been performed yet. The solution of variation leads to the determination of the optimal parylene thickness, evaluation of the systematic effects of bending, and analysis of the correlation between the transparency of the thermistor and its resistance. In addition, the high resistance of ≈1 MΩ at 25 °C was derived from the limited number of conductive paths (the number of fibers) (Figure S8, Supporting Information). One method of decreasing the readout resistance of thermistors is to reduce the spacing or increase the finger numbers of the interdigitated electrodes. However, polymer‐fiber‐templated electrode width still has been limited to a few hundred micrometers.^[^ ^8^, ^16^ ^]^ Lithography techniques are one of the promising approaches to obtain finer electrode patterns.^[^ ^41^ ^]^

Addressing the aforementioned drawbacks can pave the way for practical applications of ultrathin mesh PTC thermistors. Ultraflexible thermistors can serve as overheat prevention components for on‐skin^[^ ^5^, ^6^, ^7^, ^8^, ^9^, ^10^ ^]^ or implantable devices.^[^ ^12^, ^13^, ^14^, ^26^ ^]^

## 4 Conclusion

In conclusion, we successfully developed an ultrathin mesh‐type polymer PTC thermistor via electrospinning. The fiber‐type thermistor with a thickness of ≈5 µm exhibited an increase in the resistance by three orders of magnitude in a narrow temperature range (≈2 °C). By coating a parylene layer of sufficient thickness, the mesh thermistor served as a switch‐off function at the 400th temperature increase. The mesh structure enabled the fiber layer with a density of 16.5 µg cm^−2^ to have a light transmittance of over 90% in the 400–800 nm region. Additionally, we demonstrated an ultraflexible thermistor whose temperature characteristics were maintained when wrapped around a needle of 280 µm radius by fabricating a mesh thermistor on a 1.4 µm film. Furthermore, a gas‐permeable polymer PTC thermistor was developed by fabricating the composite fibers on a mesh substrate. The proposed ultrathin mesh polymer PTC thermistors will provide on‐skin and implantable applications with overheat prevention functions.

## 5 Experimental Section

### Synthesis of the Acrylate Polymer

Initially, 5.1 g of octadecyl acrylate (Sigma‐Aldrich), 0.9 g of butyl acrylate (Sigma‐Aldrich), 0.06 g of 2,2‐dimethoxy‐2‐phenylacetophenone (Sigma‐Aldrich), and 1.5 g of THF (FUJIFILM Wako Pure Chemical Corporation) were mixed. 0.05 wt% PEDOT‐TMA (in nitromethane, Sigma‐Aldrich) was added to this mixture. When 0.06, 0.15, 0.30, and 0.60 g of PEDOT‐TMA solutions were introduced into the monomer solution, they corresponded to the final PEDOT‐TMA concentrations of 0.005, 0.015, 0.025, and 0.050 wt%, respectively, for the polymer. Before adding, the PEDOT‐TMA solution was alternately stirred three times using a vortex mixer (VTX‐3000L, LMS) and sonicated for 3 min using an ultrasonic cleaner (AS482, AS ONE). The mixed solution was stirred for more than 2 h at >1000 rpm using a magnetic stirrer (HPS‐100T, AS ONE) with wrapping aluminum foil. The solution was then irradiated with UV light for 2 h using an 8 W UV lamp (UVL‐28 EL Series UV Lamp, Funakoshi) at a wavelength of 365 nm for polymerization. After UV irradiation, the polymer was stored overnight in a vacuum dryer (VO‐FR1, AS ONE). Furthermore, the polymer was placed in an oven (DKN302, Yamato Scientific) and heated at 80 °C for 1 h to remove the residual solvent.

### Mesh Substrate Preparation

The mesh substrate was fabricated via electrospinning.^[^ ^38^ ^]^ First, a 10 wt% PVA (Wako Chemical Industries, 160–08295) aqueous solution was prepared and loaded into a 5 mL plastic syringe (Norm‐Ject, Henke Sass Wolf). Second, PVA fiber sheets were fabricated with an electrospinning machine (NANON‐03, MECC Co., Ltd.). The fibers were deposited on a rotating drum (20 cm both in diameter and in length) wrapped with a silicone‐coated paper (Toyo Aluminum Ekco Products). The distance between the needle tip (22 gauge, Terumo) of the syringe and collector was 15 cm. The drum was rotated at 100 rpm and the syringe was moved from side to side over 20 cm at 8 cm s^−1^. The applied voltage was 20 kV, and the solution was supplied at a feed rate of 0.6 mL h^−1^ for 20 min. Third, the fiber sheet was heat‐pressed between the silicone‐coated papers, which were used for delaminating the fiber sheets after heat pressing. The heat‐press process was performed using HP‐4536A‐12 (Hashima) for 5 min, at 180 °C and 30 kPa. Finally, the mesh PVA sheet was transferred onto a 125 µm polyimide film (UPILEX‐125S, Ube Industries) with a window (Figure S11a, Supporting Information). This process was performed to maintain the porous structure after the parylene coating.

### Fabrication of the Interdigitated Electrodes

Interdigitated electrodes were fabricated on films or mesh substrates via vacuum deposition. The film was a 75 µm thick polyimide (UPILEX‐75S, Ube Industries) or a 1.4 µm thick PET film (Mylar 1.4 CW02, DuPont Teijin Films). The PET film was attached in advance to a polyimide film (1020RM‐S, Okamoto Industries) with an adhesive layer. This adhesive polyimide film served as a supporting substrate to ease the handling of the 1.4 µm PET film during device fabrication. 70 nm gold was deposited on films or mesh substrates through a film mask using EX‐200 (ULVAC). The interdigitated electrode was 1 cm long, and 400 µm wide with 13 fingers. The spacing between adjacent fingers was 400 µm.

### Fabrication of Mesh‐Type Thermistors

Fiber‐mesh polymer PTC thermistors were fabricated via electrospinning. Initially, 2.0 g of acrylate polymer containing 0.015 wt% PEDOT‐TMA, 0.1276 g of carbon nanofibers (PR‐25‐XT‐LHT, Sigma‐Aldrich), and 7.0 g of THF were added to a sample bottle (No. 30, 20 mL, Maruemu) and stirred using a magnetic stirrer until the polymer dissolved. This composite solution was dispersed using an ultrasonic probe (UH‐300, SMT Co., Ltd.). The dispersion was performed while cooling with ice water under the condition of 40% power and 60% pulse for ≈1 h. During sonication, the evaporation of the organic solvent adjusted polymer content to 40–42 wt% in the solution. The sonicated composite solution was immediately loaded into a plastic syringe, which was followed by electrospinning. The solution was extruded at a rate of 2.5 mL h^−1^ through a 22G needle at 20 kV; the distance between the needle and collector was 15 cm. The drum was rotated at 500 rpm, and the syringe was moved from side to side over a distance of 5 cm at a speed of 5 cm s^−1^. The drum collector was wrapped with a 50 µm thick polyimide film (UPILEX‐50S, Ube Industries). The film or mesh substrates with the interdigitated electrodes were covered with a 25 µm thick polyimide film (UPILEX‐25S, Ube Industries) with a 1.1 × 1.1 cm^2^ window and were fixed with Kapton tape (Figure S11c, Supporting Information). In the case of mesh substrates, the silicone‐coated paper was placed under the substrate to avoid breakage. The temperature and humidity inside the machine were above 28 °C and below 40%, respectively. They were controlled by a dehumidifier (F‐YC80ZPX, Panasonic) and measured using a thermo‐hygrometer (CTH‐204, CUSTOM). The fiber density was controlled by varying the electrospinning time. Finally, parylene (diX‐SR, Daisan Kasei Co., Ltd.) was coated via chemical vapor deposition using LABCOTER PDS2010 (Specialty Coating Systems). The deposited parylene thicknesses were 1 µm, 1.4 µm, and 700 nm for the 75 µm thick, 1.4 µm thick, and mesh substrates, respectively. The electrode parts used to measure the resistance of the thermistor were covered with a cut slide glass to avoid being coated with parylene (Figure S11d, Supporting Information).

### Measurement of the Electrical Resistance of Polymer PTC Thermistors

The mesh‐type thermistors were placed on a hot plate (NINOS ND‐1A, AS ONE) and fixed using Kapton tape. The plate temperature was increased at a rate of 0.33 °C min^−1^, which was recorded using the software (DAQMaster for ASONE). The electrical resistance of the thermistors was measured using a digital multimeter (34465A, Keysight) with a two‐point probe method. The measurement limit of the resistance value was 1.2 GΩ. In the case of cyclic measurements, the thermal history of the mesh thermistors was eliminated by heating the samples in an oven at 100 °C for 20 min, cooling them at room temperature, and storing them at room temperature for a minimum of 1 day before the subsequent measurement.

### Microscope Observation

The device was observed under an SEM (TM‐3030 plus, Hitachi High Technologies) at an acceleration voltage of 5 or 15 kV. The parylene‐coated fiber PTC thermistors were observed under a laser microscope (VK‐9710, Keyence) and an optical microscope (VHX‐2000, Keyence). Consolidated images were generated using the equipped software.

### Fiber Density Measurement of the Composite Fiber Layer

The density of the composite fiber layer was calculated based on the mass difference before and after spinning and the spinning area. The mass was measured using an electronic balance (MSA125P, Sartorius), and the density was obtained by dividing the mass by area (1.21 cm^2^).

### Evaluation of Fiber Layer Transparency

The transparency of the composite fiber layer was investigated as a function of areal mass density. A 1.1 × 1.1 cm^2^ fiber layer was prepared on a transparent 125 µm polyethylene naphthalate (PEN) film (Q65HA‐125, Teijin) via a 25 µm polyimide film mask. For each areal mass density, the optical transmittance in the 400–800 nm wavelength range was measured using a spectrometer (V‐570D, Jasco) at intervals of 1 nm. The pristine PEN film was used as a reference of 100% transparency.

### Bending Test of the Ultrathin Thermistors

The changes in the resistance in response to the bending of the mesh PTC thermistor on an ultrathin film were investigated. Initially, the fabricated device was first peeled from the supporting substrate. Subsequently, the device was wrapped around objects with a bending radius of 9 mm (glass bottle, vial No. 2, Toseiyoki), 6.5 mm (metal rod), 2.5 mm (plastic pipette, 231, Samco Scientific), 1 mm (toothpick), 0.42 mm (needle), and 0.28 mm (needle) in that order, and the change in resistance was measured using the digital multimeter. Because it is difficult to wrap the device around objects with a bending radius of 1 mm or lower, a thin layer of glue was applied to the object to be wrapped. The tip of the film was attached using the glue, and the film was wrapped with the opposite side pulled. Furthermore, the temperature characteristics of the thermistor were measured when wrapped around the needle with a bending radius of 280 µm (Figure 4b inset).

### Evaluation of Stretchability and Mechanical Durability with Prestretching

Mesh thermistors with wrinkled structures were fabricated using the prestretch method. First, Ecoflex (00‐30, Smooth‐On) was poured onto an acrylic adhesive sheet (LUCIACS CS9862UA, Nitto Denko Corporation) and cured by heating in an oven at 80 °C for 2 h. This resulted in the preparation of a 1.2 mm thick Ecoflex sheet with an adhesive layer on the surface. The elastomer sheet was cut into a 4 × 4 cm^2^ piece, which was fixed to a platform using Kapton tape to enable the sheet to move in only one direction. The initial distance of the stretchable part was 1.6 cm. With the sheet stretched by 4 mm (25% stretching), a mesh thermistor fabricated on an ultrathin film was attached to the sheet. When the stretching state was released, a 25% prestretched wrinkle‐structured device was obtained. The temperature characteristics of the wrinkled thermistors were investigated at 0% and 20% strains. The stretched state was fixed using the Kapton tape (Figure S10c inset, Supporting Information). To investigate the mechanical durability of the device, the change in the electrical resistance with respect to the cyclic stretching and release was measured. The device was manually stretched by 25% and released to its original state 200 times. After 200 cycles, the temperature characteristics of the thermistor at 0% strain were measured. The thermistor side faced the plate when measuring the temperature characteristics in the case of the wrinkled devices.

### Evaluation of the Water Vapor Permeability of PTC Thermistors

The water vapor transmission rate was evaluated to investigate the gas permeability of the fiber‐mesh PTC thermistor. Five glass bottles (1 cm diameter opening, vial No. 2, Toseiyoki) containing 1 g of pure water were prepared. The first bottle was uncovered (“open”); the second was covered with a parylene‐coated mesh electrode (“mesh electrode”); the third was covered with a sample that was fabricated by electrospinning composite fibers on the mesh electrode and coating with parylene (“mesh PTC”); the fourth was covered with a sample that was fabricated by mounting 25 µm thick composite film on the mesh electrode and coating with parylene (“film PTC”); and the fifth was covered with aluminum foil (“Al foil”). The samples were attached to the bottles using Araldite (Nichiban). The parylene‐coated samples were transferred in advance to a supporting frame to coat the parylene in a freestanding state. The thickness of parylene was 700 nm. These bottles were stored in a dry chamber (UVOH‐520SA, AS ONE) for 1 week at a temperature of ≈18 °C and 5% humidity, which were measured using a thermo‐hygrometer (THI‐HP, AS ONE). The subsequent change in mass was measured using an electronic balance (ITX‐120, AS ONE).

## Conflict of Interest

The authors declare no conflict of interest.

## Supporting information

Supporting Information

## Data Availability

The data that support the findings of this study are available from the corresponding author upon reasonable request.
