# Supplementary material for: Ultrathin Fiber‐Mesh Polymer Thermistors
Source: Adv Sci (Weinh). 2022 Sep 4;9(30):2202312. doi: 10.1002/advs.202202312 (PMC9596841; doi:10.1002/advs.202202312)
Supplement: Supplementary file 1 — Supporting Information [file ADVS-9-2202312-s001.pdf]

Supporting Information

Ultrathin fiber-mesh polymer thermistors

Chihiro Okutania, Tomoyuki Yokota, Takao Someya**

Supplementary Information Text

Supplemental Note 1.

Initially, we investigated the effect of PEDOT-TMA on the fiber diameter of the acrylate polymer. The polymer fibers were fabricated via electrospinning and observed using SEM (Figure S2a). The fiber diameter of the conventional acrylate polymer without PEDOT-TMA was 14.6 ± 4.1 µm (Figure S2b,c). The diameter of the polymer fibers varied based on the amount of PEDOT-TMA. The smallest diameter of 6.4 ± 0.8 µm was obtained when the amount of PEDOT-TMA was 0.015 wt%; the addition of more than 0.015 wt% PEDOT-TMA increased the fiber diameter.

Subsequently, we investigated the effect of PEDOT-TMA on the polymer temperature characteristics. The thermal properties of the polymers were investigated using differential scanning calorimetry (DSC). Minor changes were observed in the heat flow when PEDOT-TMA was less than 0.050 wt% (Figure S3a). Additionally, the melting point calculated by DSC was approximately 39 °C (Figure S3b).

The crystallinity of the acrylate polymer containing 0.015 wt% PEDOT-TMA at each temperature was investigated using X-ray diffraction (XRD) (Figure S3c,d). The peak changed slightly from 21.5° to 21.4° when the polymer was heated from 30 to 35 °C. The peak of this point was not observed at 42 °C, which was beyond the melting point of the polymer (Figure S3b). The 21.5° peak was observed again when the polymer was cooled from 42 to 30 °C.

We further investigated the effects of the additional PEDOT-TMA on the thermal properties of the polymer PTC thermistors. We evaluated the printed film thermistors that were fabricated using acrylate polymer, graphite, and THF (Figure S4a). Regardless of the amount of PEDOT-TMA, the changes in the resistance of the thermistors were approximately five orders of magnitude, indicating that the effect was minor (Figure S4b). In addition, little difference in the change in thermistor characteristics was observed until at least 100 cycles (Figure S4c). Therefore, we used an acrylate polymer containing 0.015 wt% PEDOT-TMA, which exhibited the smallest fiber diameter, to fabricate the fibrous thermistor.

Supplemental Note 2.

Carbon nanofibers were selected as conductive fillers to fabricate the fibrous thermistors owing to their one-dimensional structures, which facilitates the fabrication of one-dimensional structured fibers. We fabricated film-type thermistors using a printing process and evaluated their characteristics with different amounts of conductive fillers to determine the ideal amount of conductive fillers for the device operation (Figure S5a). By increasing the amount of conductive fillers, the electrical resistance at 25 °C decreased, and the resistance change ratio (R_{max}/R_0) also decreased (Figure S5b). A large amount of the conductive material caused clogging during electrospinning, which generated beads owing to the aggregation of conductive fillers. Based on these results, we set the amount of conductive fillers to be 6 wt%.

Furthermore, we investigated the thermal cyclic characteristics of thermistors containing 6 wt% carbon nanofibers. When the temperature increased in the second and third cycles, both melting points were 38.4 °C. This indicates that the composite material exhibited repeatable temperature characteristics (Figure S5c).

Supplemental Materials and Methods

Preparation of pristine acrylate polymer fibers and measurement of their diameters: The fiber diameters of the acrylate polymer were investigated considering each PEDOT-TMA amount. The acrylate polymer and THF were mixed in a mass ratio of 2:3 to obtain a 40 wt% polymer solution. Polymer fibers were fabricated on a gold-coated film via electrospinning under an applied voltage of 20 kV, and a feed rate of 0.6 mL h^{-1} with a syringe-collector distance of 15 cm. The morphologies of the fibers were observed using SEM at an acceleration voltage of 5 kV. The diameters of more than 50 fibers measured manually. The results were expressed as the mean and standard deviation.

DSC measurement: The effect of PEDOT-TMA on the temperature properties of the acrylate polymer was investigated using DSC (DSC-60Plus, Shimadzu). 5.0 mg of acrylate polymer containing different amounts of PEDOT-TMA was packed in an aluminum pan. 10.0 mg of alumina (Al_2O_3 powder, Shimadzu) was packed in an aluminum pan as the reference. The sample was first heated to 100 °C and then cooled to 15 °C to eliminate the thermal history. Subsequently, the sample was heated from 15 to 60 °C and cooled from 60 to 15 °C at 5 °C min^{-1}, and the heat flow was measured.

The thermal characteristics of the polymer PTC thermistor with carbon nanofibers were investigated using DSC. 5.0 mg of PTC thermistor (PEDOT-TMA concentration: 0.015 wt%, containing 6 wt% carbon nanofiber) was packed in an aluminum pan and DSC measurements were performed. The sample was cooled to −5 and then heated to 150 °C; this process was performed three times. The heat flow was measured by setting the temperature change rate to 5 °C min^{-1}. The DSC measurements were performed under a nitrogen gas flow rate of 30 mL min^{-1}. The melting points of the materials were calculated using software (LabSolutions TA, Shimadzu).

XRD analysis. The crystallinity of the acrylate polymer was measured at different temperatures via XRD (Smart Lab, Rigaku) using the out-of-plane method. The polymer containing 0.015 wt% PEDOT-TMA was prepared on a 24 mm × 24 mm glass. The temperature was varied in the order 30, 35, 37, 39, 42, and 30 °C, and the crystallinity was measured at each temperature. The XRD patterns were measured every 0.1° using Cu-Kα radiation (λ = 1.5418 Å) in the range of 15 to 30°.

Fabrication of the graphite-containing film thermistors for PEDOT-TMA evaluation: The temperature characteristics of the polymer PTC thermistors were evaluated at each PEDOT-TMA concentration using film-type thermistors with graphite. For the evaluation, the polymer PTC ink was prepared by mixing 1.46 g of polymer, 0.54 g of graphite (2–3 µm size, Furuuchi Chemical Corporation), and 0.2 g of THF using a planetary centrifugal vacuum mixer (ARV-310, Thinky Corporation) at 2000 rpm for 5 min; the mixing was repeated three times. The ink was placed on a hot plate at 60 °C to reduce its viscosity before using the mixer. The 25-µm-thick film-type thermistors were fabricated by printing on interdigitated electrodes using a 25-µm-thick polyimide film mask. The substrate was a 75 µm polyimide film, and the printing was performed on a hot plate at 60 °C. After the printing process, the thermistor was annealed at 100 °C for 1 h to remove the residual solvent. Finally, 1.4 µm parylene was coated via chemical vapor deposition. The electrodes used to measure the resistance of the thermistor were covered with a glass slide to avoid being coated with parylene.

Fabrication of film thermistors with different amounts of carbon nanofibers: The ultrasonic treatment was used to disperse 2 g of acrylate polymer with 0.015 wt% PEDOT-TMA, different amounts of carbon nanofibers, and 7 g of THF. The amounts of carbon nanofibers of 0.083, 0.105, 0.128, 0.151,

and 0.174 g corresponded to 4, 5, 6, 7, and 8 wt% content of carbon nanofibers, respectively. The ultrasonic dispersion was performed under 40% power and 60% pulse for 1 h. The composite solution was placed in a fume hood for a day and heated in an oven at 60 °C for 1 h to remove the solvent. Subsequently, the composite material was heated in an oven at 60 °C, and it was mixed once using the planetary centrifugal vacuum mixer at 2000 rpm for 5 min. Thermistors with a thickness of 25 µm were fabricated using the same printing process as described above. After the printing process, the thermistor was heated to 100 °C for 1 h. Parylene was not coated on the film-type thermistor that contained carbon nanofiber.

Measurement of electrical resistance of film-type thermistors: The resistance of film-type thermistors was measured similar to that of the mesh-type thermistors (described in Materials and Methods section of the main text). R_0 denoted the resistance at 25 °C, and R_{max} indicated the maximum resistance during the measurement. In the case of cyclic measurements, the thermal history was eliminated by storing the thermistors at room temperature for a minimum of one day before the subsequent measurement.

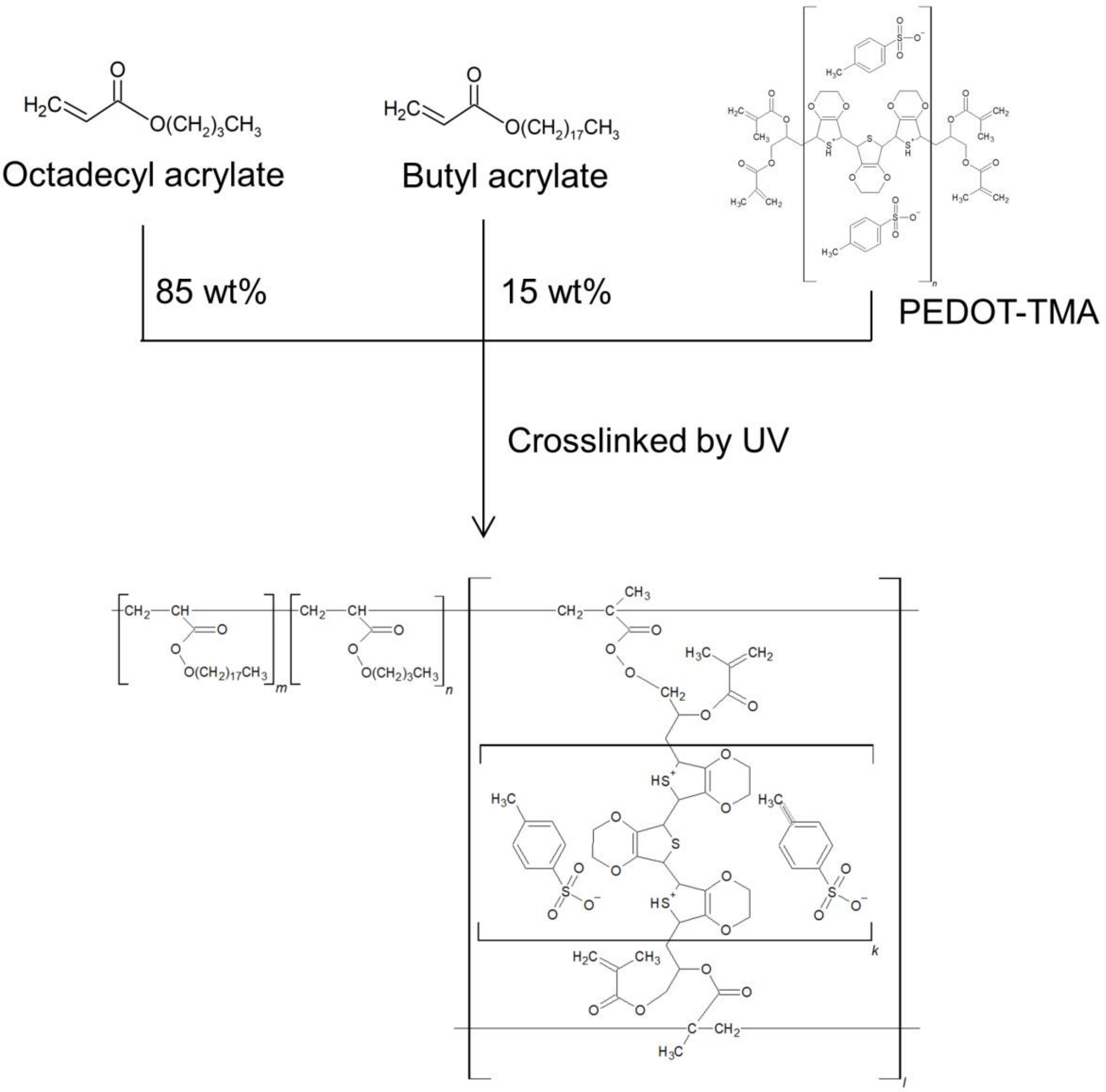

Figure S1. Synthesis process of acrylate polymer with poly(3,4-ethylenedioxythiophene)-tetramethacrylate (PEDOT-TMA).

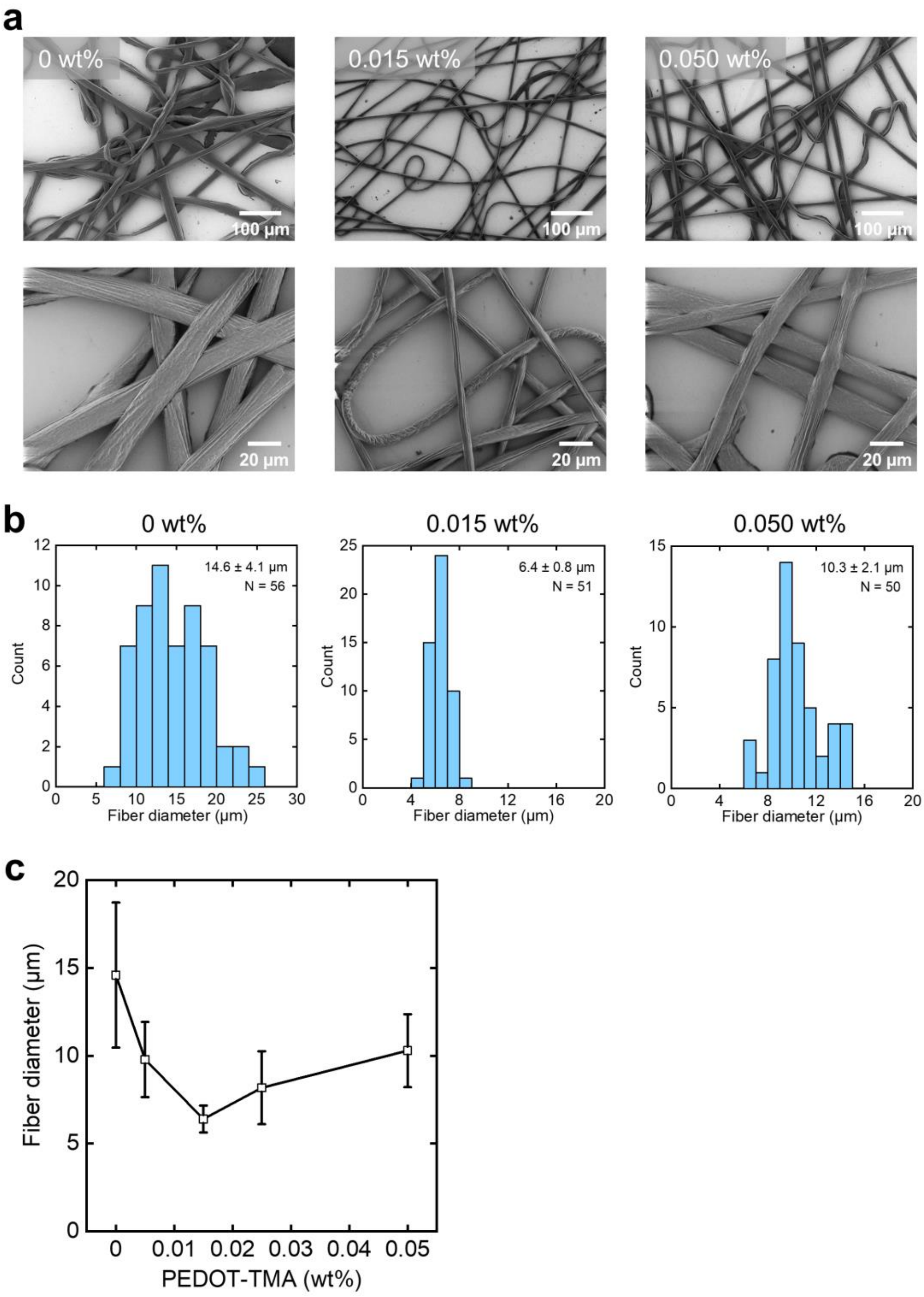

Figure S2. Effect of poly(3,4-ethylenedioxythiophene)-tetramethacrylate (PEDOT-TMA) on the fiber diameter of acrylate polymer. a) Scanning electron microscope (SEM) images of polymer fibers with 0, 0.015, and 0.050 wt% PEDOT-TMA concentrations. b) Histograms of the fiber diameter with 0 wt%, 0.015 wt%, and 0.050 wt% PEDOT-TMA concentration, respectively. Numbers on the top-right corner

indicate the mean ± standard deviation of the fiber diameter and sample numbers (N). c) Fiber diameter as a function of PEDOT-TMA concentration. Error bars describe the standard deviation.

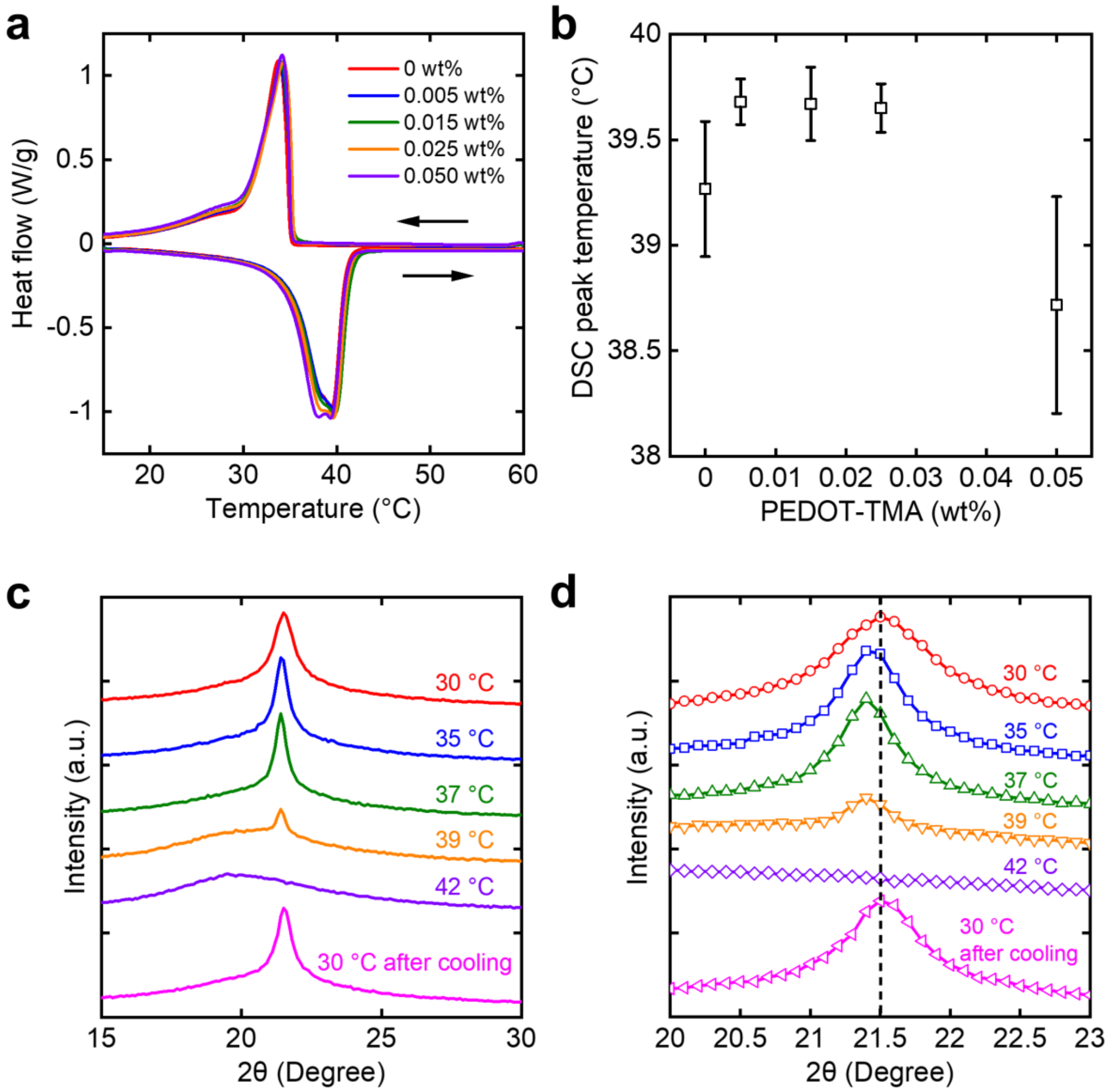

Figure S3. Effect of poly(3,4-ethylenedioxythiophene)-tetramethacrylate (PEDOT-TMA) on material characteristics. a) Differential scanning calorimetry (DSC) curve of acrylate polymer for different PEDOT-TMA concentrations. b) Melting point of the acrylate polymer as a function of PEDOT-TMA concentration (N = 3). Error bars describe the standard deviation. c) Out-of-plane X-ray diffraction (XRD) spectrum of acrylate polymer containing 0.015 wt% PEDOT-TMA at each temperature. d) Magnification of (c) in the range of 20–23°.

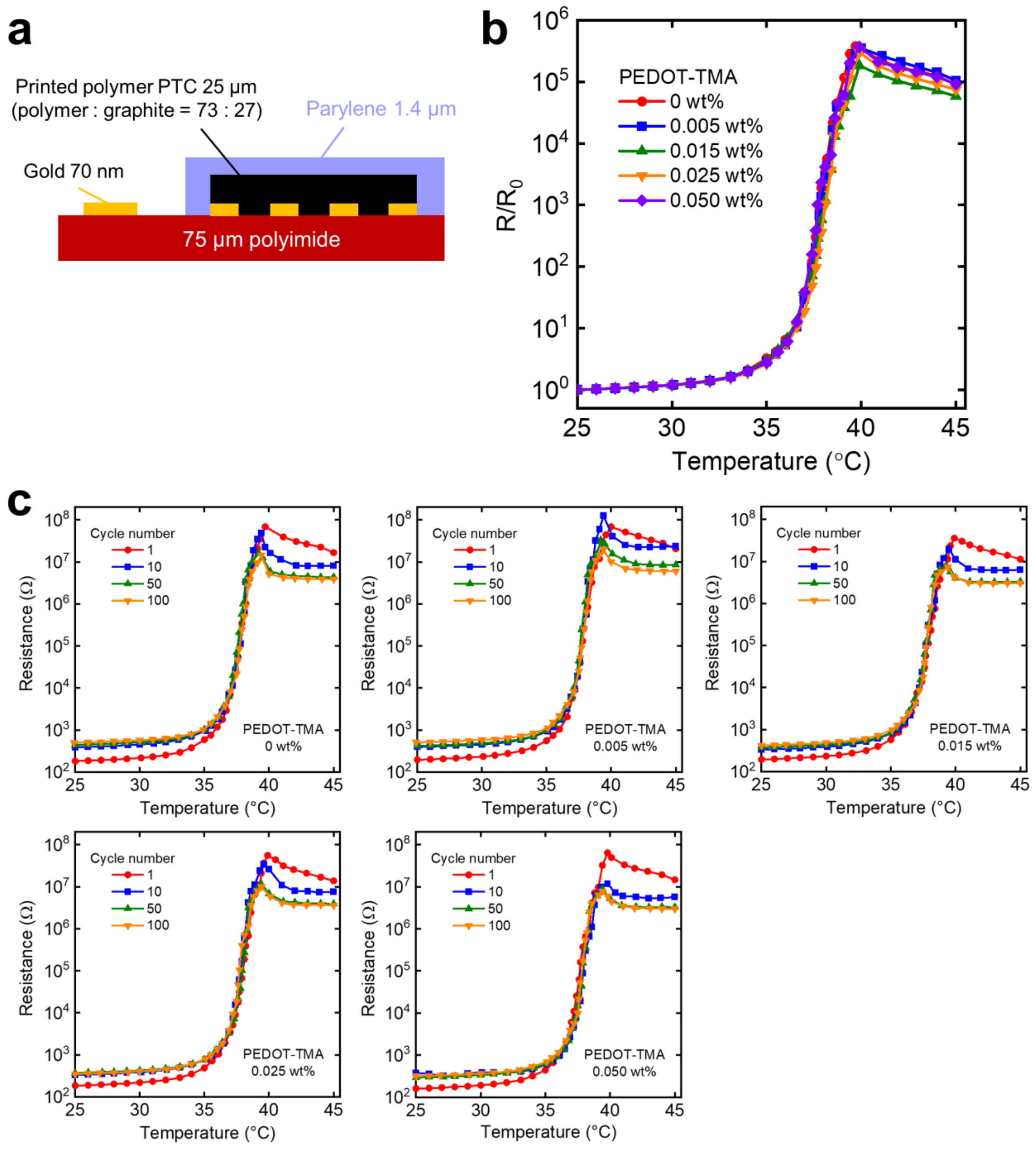

Figure S4. Effect of poly(3,4-ethylenedioxythiophene)-tetramethacrylate (PEDOT-TMA) on film-type polymer positive temperature coefficient (PTC) thermistors with graphite. a) Schematic cross-sectional device structure. b) Change in the relative resistance of the thermistor as a function of temperature for different PEDOT-TMA concentration. c) Thermal cyclic characteristics of the thermistor at each PEDOT-TMA concentration.

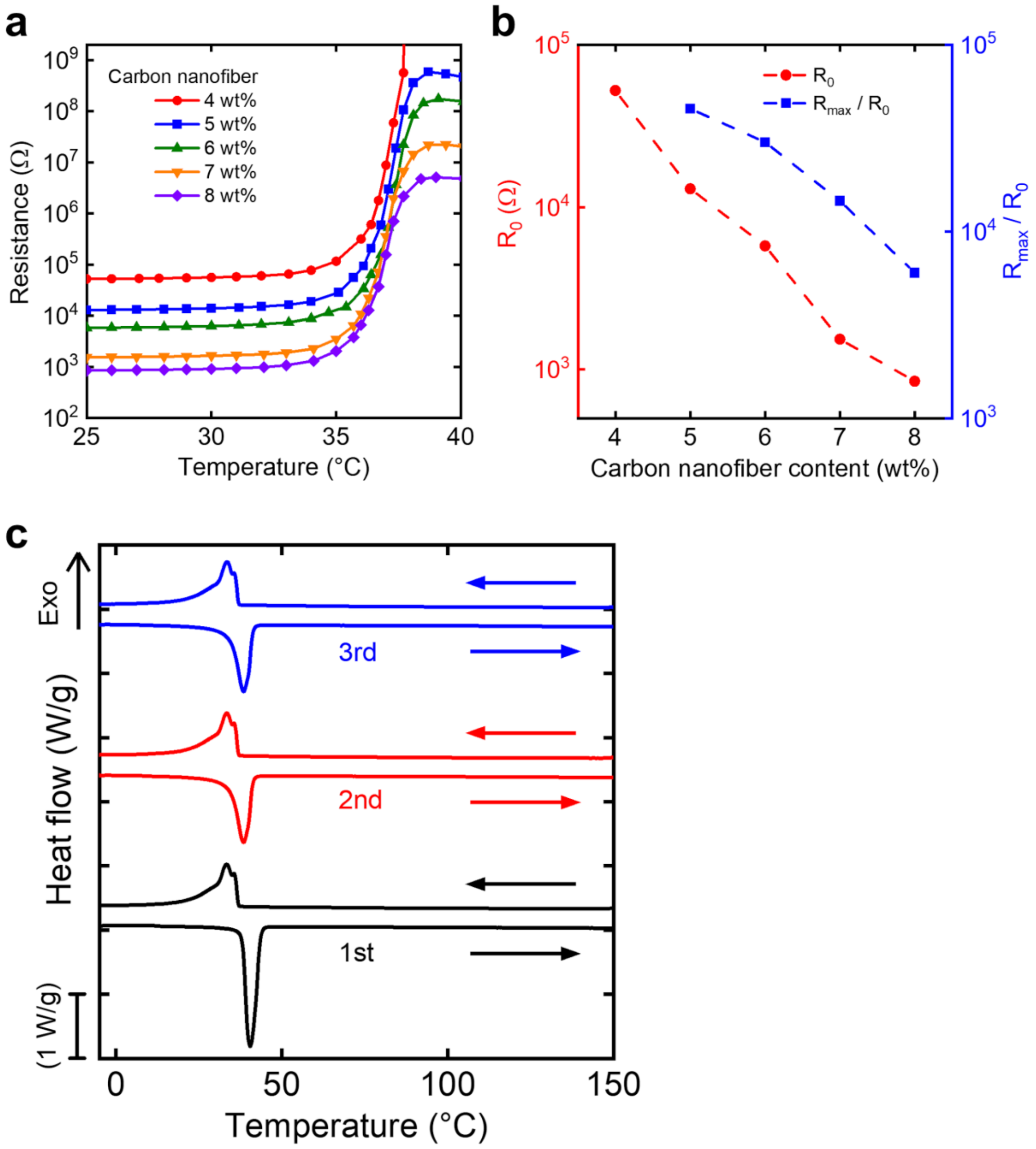

Figure S5. Film-type polymer positive temperature coefficient (PTC) thermistor with carbon nanofiber. a) Change in the resistance as a function of temperature for each carbon nanofiber concentration. b) Resistance at 25 °C (R_0) and the ratio of the maximum resistance (R_{max}) to R_0 as a function of carbon nanofiber content. R_{max}/R_0 at 4 wt% carbon nanofiber could not be evaluated. c) Differential scanning calorimetry (DSC) curve of the composite material (6 wt% carbon nanofiber) in three heating–cooling cycles.

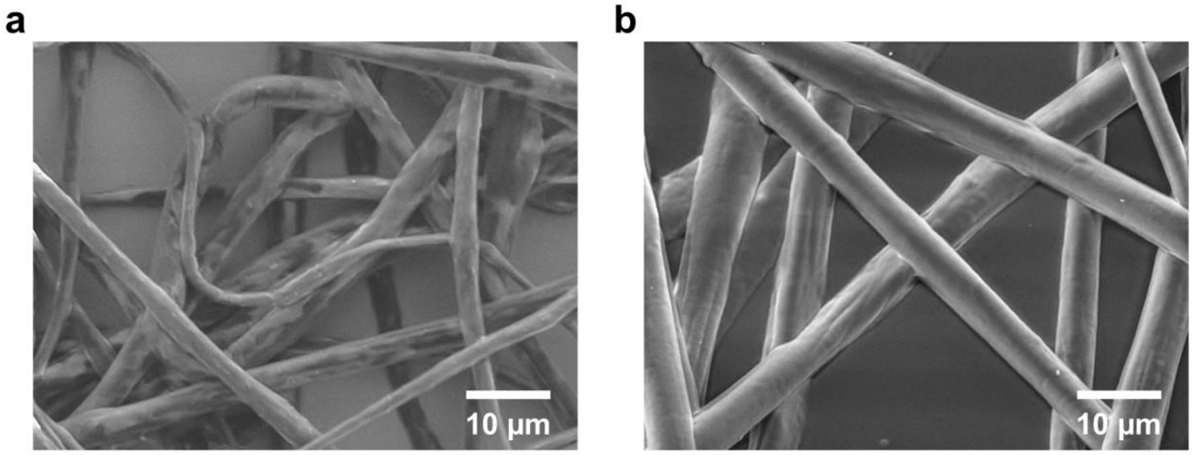

Figure S6. Scanning electron microscope (SEM) images of composite fibers. Fiber morphologies (a) before parylene coating and (b) after 1-µm-thick parylene coating.

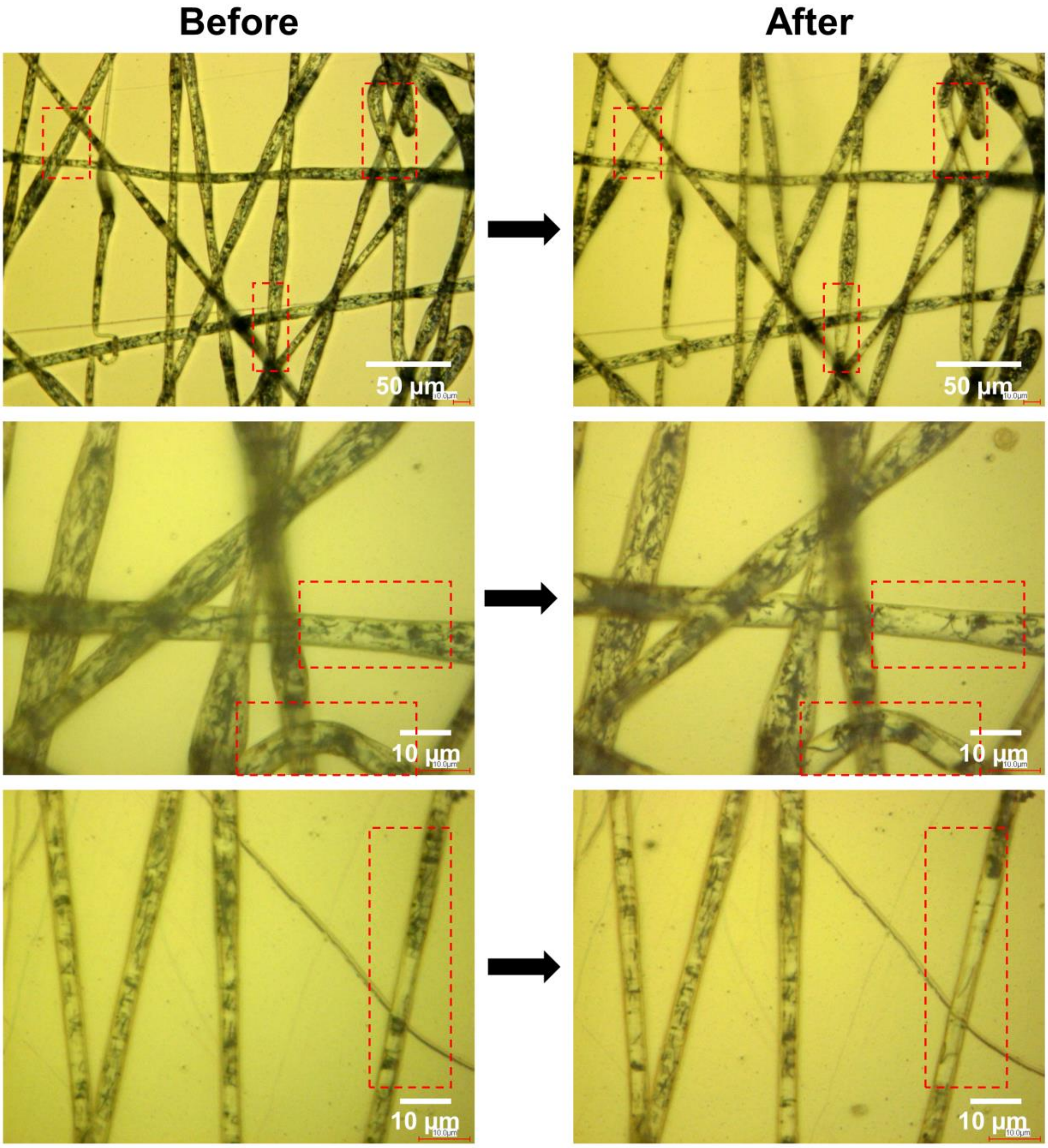

Figure S7. Optical images of composite fibers before and after the thermal cyclic tests. Conductive fillers in red dashed rectangles moved significantly before and after the cycles.

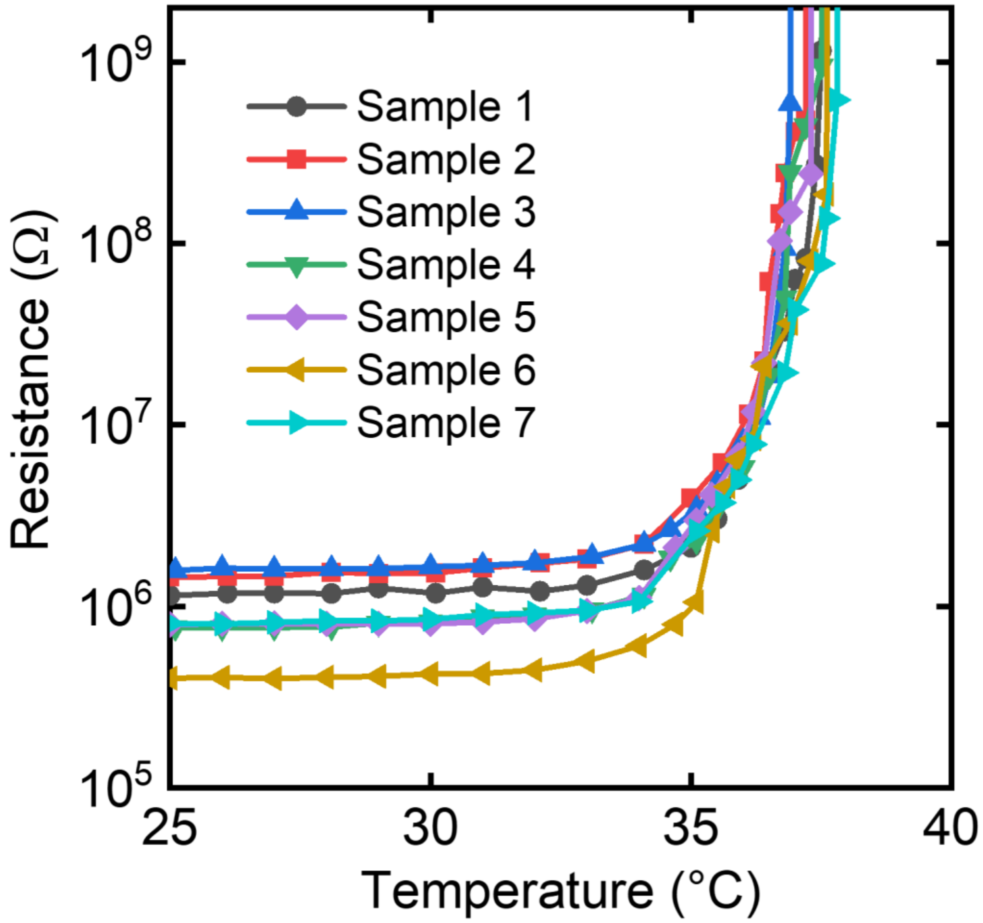

Figure S8. Variation in device characteristics with simultaneous fabrication. The electrospinning time was 20 s. Sample 1 was identical to the sample with a density of 16.5 μg cm^{-2} in Figure 3b

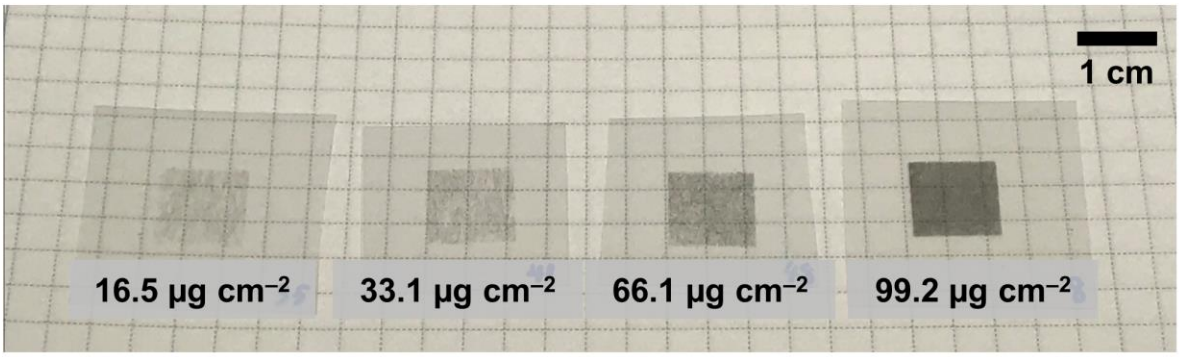

Figure S9. Photograph of a composite fiber layer for fiber density. Composite fibers with a density of 16.5, 33.1, 66.1, and 99.2 μg cm^{-2} were deposited on transparent polyethylene naphthalate (PEN) films.

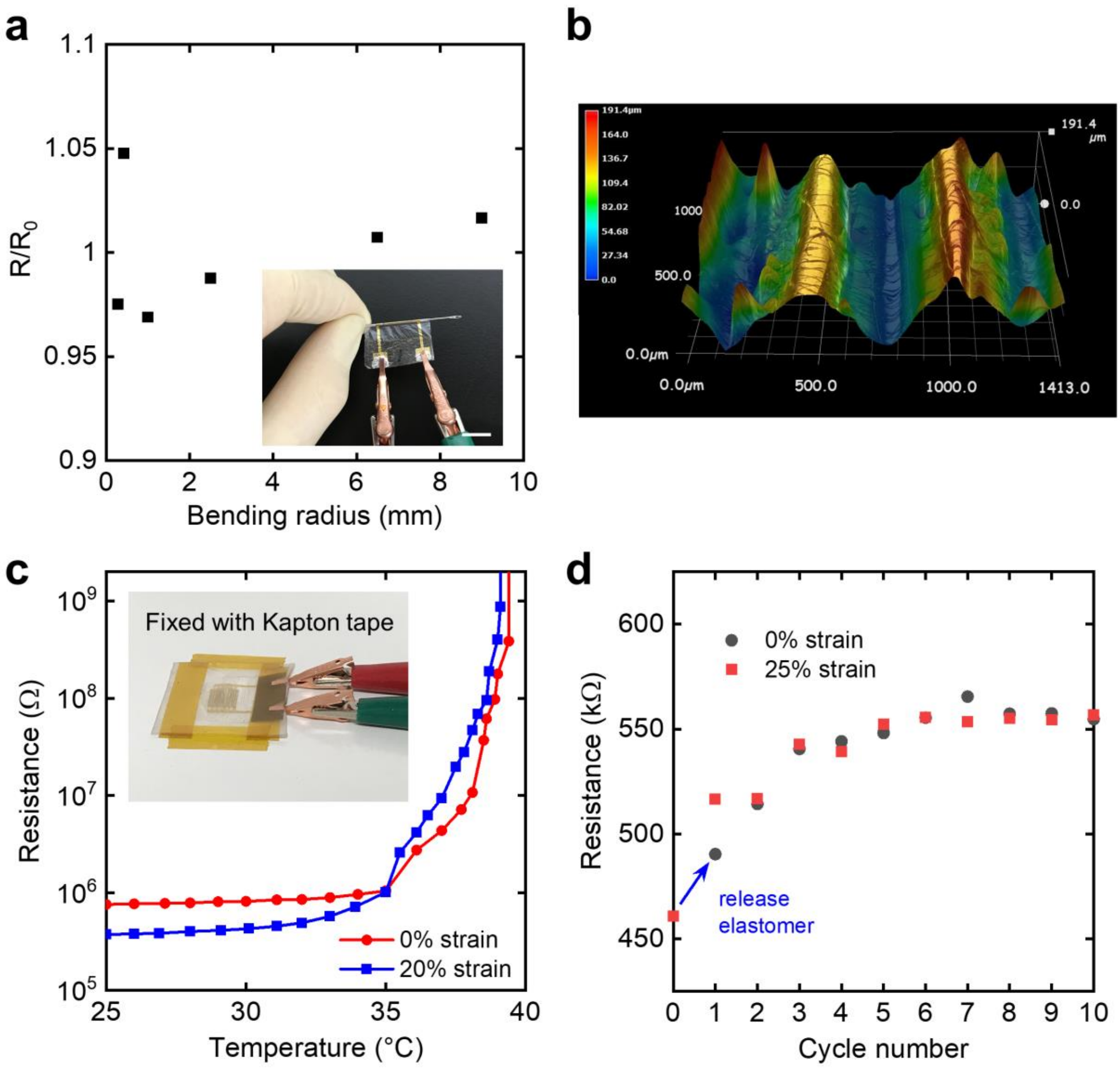

Figure S10. Mechanical and thermal characteristics of the mesh thermistor on an ultrathin substrate. a) Dependence of the relative change in resistance of mesh thermistor on the ultrathin film substrate based on its bending radius. Inset: A photograph of the thermistor wrapped around a needle with a radius of 280 µm. b) Wrinkle topography of the thermistor characterized by a three-dimensional optical microscope. The color map indicates the height of the wrinkle device. c) Change in the resistance of the wrinkled thermistor at 0 and 20% strains. Inset: A photograph of the wrinkled thermistor at 0% strain during measurement. d) Change in the resistance at 10 stretching cycles (Magnification of Figure 4e).

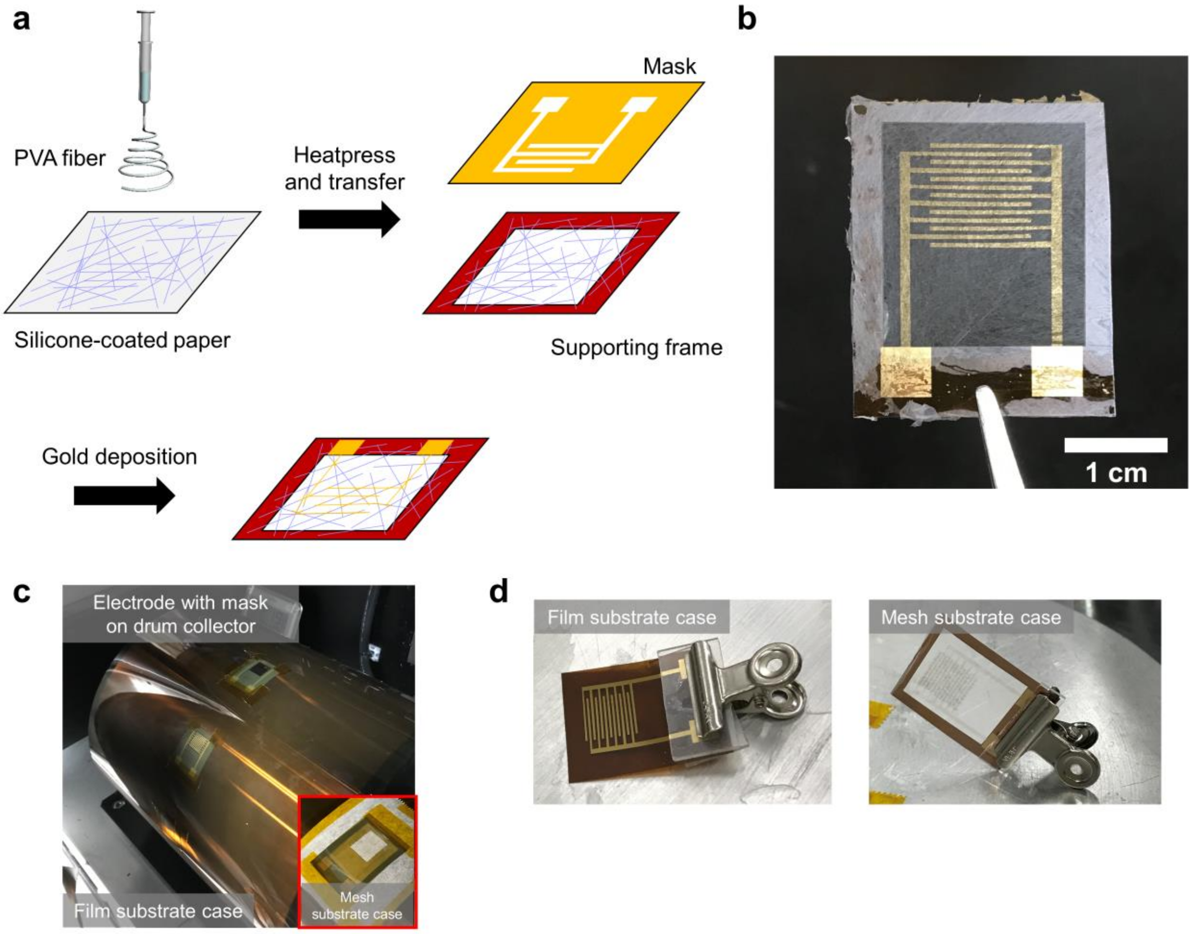

Figure S11. Fabrication of the gas-permeable thermistor. a) Schematic of the fabrication process for mesh substrate with patterned electrodes. b) A photograph of the interdigitated electrode on polyvinyl alcohol (PVA) mesh substrate with a supporting frame. c) A photograph of electrodes on film and mesh substrates covered with a film mask before the electrospinning process. d) Photographs of a fibrous thermistor on a film and mesh substrate for the parylene coating process.
